# Federated analytics for non-communicable disease surveillance in the European health data space: a scoping review and conceptual framework

**DOI:** 10.3389/fpubh.2026.1911992

**Published:** 2026-08-21

**Authors:** Fabrizio Carinci, Stephen Fava, Iztok Štotl, Nicholas Nicholson

**Affiliations:** 1 Unicamillus International Medical University, Rome, Italy; 2 Department of Medicine, Mater Dei Hospital of Malta, Msida, Malta; 3 Department of Endocrinology, Diabetes and Metabolic Diseases, University Medical Center Ljubljana, Ljubljana, Slovenia; 4 Faculty of Medicine, University of Ljubljana, Ljubljana, Slovenia; 5 Joint Research Center (JRC), European Commission, Ispra, Lombardy, Italy

**Keywords:** distributed regression, distributed statistical inference, EHDS, epidemiology, federated analytics, NCD surveillance, observational health data, privacy-preserving analytics

## Abstract

**Background:**

The growing burden of non-communicable diseases (NCDs) in Europe has intensified the need for timely, comparable, and policy-relevant health indicators derived from increasingly heterogeneous health data ecosystems. The European Health Data Space (EHDS) represents a major policy initiative to facilitate the secondary use of health data while preserving privacy, security, and national data sovereignty. In this context, federated analytics can foster international comparisons without requiring the transfer of person-level data.

**Objectives:**

This scoping review aimed to map federated analytical approaches relevant to the production of NCD indicators within the EHDS and to develop a conceptual framework linking distributed statistical methods, privacy-preserving infrastructures, and policy-oriented surveillance requirements.

**Materials and methods:**

A scoping review was conducted following the PRISMA Extension for Scoping Reviews. Searches were structured into three complementary conceptual domains: (a) federated analytical approaches, (b) distributed statistical inference methods, and (c) governance and health-data infrastructures relevant to the EHDS. Searches were performed in PubMed, Scopus, and IEEE Xplore, for studies published between 2010–26. Records were exported with abstracts and full bibliographic metadata. Deduplication and metadata-aware merging were conducted across databases using DOI and normalized-title matching.

**Results:**

We retrieved a total of 2,362 records, of which 1,285 were unique records and 104 were finally retained. The literature revealed a heterogeneous but rapidly expanding ecosystem of distributed analytical approaches. We identified three major domains: (1) distributed regression; (2) federated or distributed analytical infrastructures; and (3) privacy-preserving federated epidemiological analysis.

**Discussion:**

An increasing range of solutions for federated analytics is available for policy-grade NCD indicators. Longitudinal and survival models remain methodologically complex because of covariance structures and globally coupled risk sets. A modular approach is needed to incorporate public-health intelligence and a dynamic set of interoperable tools in the EHDS, with the active support of a decentralized network of active stakeholders.

**Conclusion:**

Federated analytics is shifting the paradigm from centralized data pooling to computation-to-data architectures. The successful implementation of NCD surveillance in the EHDS require integrating statistics with legal, IT, policy and governance mechanisms. The review outlined a conceptual framework that can couple technical innovation with the best architecture for public-health knowledge production.

## 1 Introduction

Non-communicable diseases (NCDs), including cardiovascular diseases, cancer, diabetes, chronic respiratory diseases, and neurodegenerative disorders, remain the leading causes of mortality, morbidity, disability, and healthcare expenditure across Europe (1).

Effective prevention and management of NCDs depend increasingly on the availability of robust and timely health indicators capable of supporting surveillance, benchmarking, risk stratification, healthcare planning, and policy evaluation (2). Such indicators include prevalence and incidence measures, mortality rates, survival estimates, multimorbidity indices, quality-of-care metrics, avoidable hospitalization rates, and socioeconomic inequalities in access and outcomes (3).

The production of NCD indicators increasingly relies on large-scale digital health ecosystems composed of electronic health records (EHRs), administrative databases, disease registries, claims data, wearable technologies, and population-based observational cohorts (4, 5). These developments have transformed the methodological landscape of epidemiological research and public-health surveillance by enabling increasingly large-scale analyses of heterogeneous populations across institutions and jurisdictions (6).

Historically, centralized pooling of individual-level data has represented the dominant paradigm for multicentric epidemiological research and public-health surveillance. However, growing concerns regarding compliance with the General Data Protection Regulation (GDPR) (7) have rendered large-scale centralization increasingly difficult to operationalise (8). Cross-border data transfer is still hampered by administrative, political and often incompatible institutional data governance highlighted before the GDPR (9).

Federated analytics (FA) has consequently emerged as a promising paradigm for distributed health data analysis (10, 11). Broadly defined, FA encompasses computational strategies in which statistical estimation is performed across decentralized datasets through the exchange of intermediate analytical quantities rather than raw individual-level data. Such approaches include distributed regression, federated optimisation, secure aggregation protocols, privacy-preserving query systems, and cryptographic methods. Although frequently discussed alongside federated learning, FA encompasses a more focused set of statistical tasks that can be ideal to support EU applications (12, 13).

The European Health Data Space (EHDS) represents a potentially transformative development in this context (14). The EHDS aims to facilitate primary and secondary uses of health data across Europe while preserving privacy, security, interoperability, and the autonomy of national data governance. Pursuing these objectives will likely benefit from FA across different jurisdictions.

The conceptual foundations of FA for the continued production of public health indicators preceded their recent explosion of at least two decades, when a series of projects were funded by the EU as part of the health information strand (15). The technological innovation emerged in parallel with the establishment of international registry networks (5) offered new tools that can be instrumental for implementing NCD surveillance in the EHDS.

Subsequent methodological developments included operational frameworks and non-disclosive federated architectures for pooled epidemiological analyses (16), distributed analytical paradigms for generalized linear models (17), logistic regression (18) and survival analysis (19).

In 2022, the Commission's Directorate-General of Health and Food Safety (DG SANTE) and the Joint Research Center (JRC) launched the “Collaborative Health Information European Framework” (CHIEF) to collect new ideas and solutions for the regular collection of NCD indicators, through the formation of an international expert group focusing on three main areas: metadata, federated analytics, and stakeholder engagement (20).

The rapid growth of FA literature was noted by the expert group, raising questions on the range and applicability of different approaches in real-world settings.

Firstly, it was noted that the methodological domains were still highly heterogeneous, including machine-learning and artificial intelligence (AI) that are usually quite distant from traditional epidemiological analyses. Secondly, existing reviews disproportionately emphasized technical architectures that are less relevant for policy and planning in the EU regulatory context (21–23). Thirdly, relatively limited attention was devoted to the direct use of FA for public health surveillance and policy-grade indicator production within the EHDS.

For this reason, it was agreed to carry out a scoping review, with the following aims:

- To map federated analytical approaches applicable for the production of NCD indicators.
- To examine methodological developments relevant to public health policies.
- To develop a conceptual framework for the application of federated analytics in the EHDS.

## 2 Materials and methods

### 2.1 Rationale of the methodology

Two main questions regarding FA approaches emerged within CHIEF as subjects of interest:

- which methods should be used for the timely computation of risk-adjusted indicators.
- what are the solutions applicable in the context of international organizations.

Regarding risk-adjusted indicators, fair international comparisons require accurate information on the number of outcomes (numerators) among persons at risk (denominators), adjusting for potential confounders to control for bias induced by imbalanced case-mix of risk factors and comorbidities. In this context, data processing operations that are considered straightforward in large databases, may be complex to perform in a federated infrastructure. For instance, survival analysis for cancer indicators (24) may not be easily applied without person-level data. Similarly, longitudinal models e.g. those used in diabetes to monitor cardiovascular risk (6) or amputation-free survival (25) may require specific methods in a federated network. Therefore, this scoping review targeted papers related to the broad spectrum of areas including FA, privacy preserving mechanisms, governance infrastructure and federated learning. On the other hand, we discarded methods that specifically explore complex relations e.g. machine learning, large language models and generative/explainable AI.

Related to the applicability in international organizations, we aimed to apply a structured search to extract validated solutions that could operate in a semi-automated way within the EHDS. For example, the OECD hospital performance project designed a two-step global data collection to benchmark quality indicators. The process required continuous refinement of the method and extensive collaboration with national governments and international experts, which may not be sustainable long term (26). For this reason, the review targeted infrastructures, methods, applications and networks capable of devolving the use of advanced statistical methods, at least partly and whenever possible, to data holders of collaborating networks. Such definition allowed to incorporate solutions that can occasionally share person-level data, but more often rely upon aggregate results. Privacy-preserving solutions targeting individual data processing e.g. blockchain or cloud data sharing were considered out of scope.

### 2.2 Study design

This review followed the PRISMA Extension for Scoping Reviews (PRISMA-ScR) (27). Considering the overlap of terms used in FA across different disciplines, the search strategy was organized into three complementary conceptual domains:

1. Federated analytical approaches.
2. Distributed statistical inference methods.
3. Governance and health-data infrastructures relevant to the EHDS.

This structure was designed to maximize sensitivity for the targeted literature, according to the rationale explained above.

Database-specific strategies combined controlled vocabulary and free-text terms related to: federated analytics, distributed statistical inference, regression, survival analysis, longitudinal modeling, epidemiology, privacy-preserving analytics, governance, interoperability, health-data infrastructures.

Reference terms included: “federated analytics,” “distributed statistical inference,” “distributed regression,” “distributed survival analysis,” “privacy-preserving analytics,” “European Health Data Space”. Further terms included the following acronyms that were widely represented in the relevant literature: “DataSHIELD,” “ODAL,” “WebDISCO,” “OHDSI,” “OMOP” (see below for full name explanations).

The full search strategy applied in the scientific search engines is reported in Table 1.

**Table 1:** Search strategy.

Block a)—Federated analytics core
TITLE-ABS-KEY( (“federated analytics” OR “federated analysis” OR “federated statistical” OR “federated statistics” OR “federated epidemiology” OR “federated regression” OR “federated survival” OR “federated Cox” OR “federated generalized linear” OR “privacy-preserving analytics” OR “privacy-preserving analysis”) AND (health OR healthcare OR clinical OR epidemiology OR “public health” OR “electronic health record” OR EHR OR surveillance OR observational) ) AND PUBYEAR > 2009
**Block b)—Distributed statistical inference**
TITLE-ABS-KEY( (“distributed statistical inference” OR “distributed regression” OR “distributed Cox” OR “distributed survival analysis” OR “distributed generalized linear model” OR “distributed logistic regression” OR “distributed longitudinal” OR “distributed estimating equation” OR “surrogate likelihood” OR “one-shot distributed” OR ODAL OR WebDISCO OR DataSHIELD) AND (health OR healthcare OR clinical OR epidemiology OR “public health” OR “electronic health record” OR EHR OR observational) ) AND PUBYEAR > 2009
**Block c)—EHDS/governance/infrastructure**
TITLE-ABS-KEY( (“European Health Data Space” OR EHDS OR “health data space” OR “common data model” OR OHDSI OR OMOP OR DataSHIELD OR “distributed health data network”) AND (governance OR interoperability OR privacy OR surveillance OR analytics OR “secondary use”) ) AND PUBYEAR > 2009

Searches were conducted using the same set of three blocks, reflecting the above listed conceptual domains, in the following engines: PubMed, Scopus, IEEE Xplore.

Searches covered papers published in scientific journals in the period from January 2010 through May 2026.

In addition to database searches, targeted manual retrieval and citation chaining were conducted for relevant European experiences incompletely captured by modern FA terminology.

This additional retrieval process ensured conceptual continuity between earlier infrastructures, such as those emerging from European registry and surveillance initiatives, and more recent FA ecosystems.

### 2.3 Data extraction and selection process

Records were exported with abstracts and full bibliographic metadata. Metadata-aware deduplication and enrichment were performed across databases using DOI matching and normalized-title similarity.

For duplicate clusters, the richest available metadata were retained, including: longest abstract, DOI, keywords and source provenance. All unique records extracted were saved in Excel spreadsheets.

The selection procedure was carried out by the first author of this scoping review, classifying and selecting the studies from a reduced version of the complete spreadsheets, blinded to the authors involved in each paper.

Studies were classified according to the following major conceptual domains:

- Federated or distributed analytical infrastructures.
- Distributed regression, including survival and longitudinal modeling.
- Governance or interoperability infrastructures supporting federated analysis of health data.
- Privacy-preserving federated epidemiological analysis.
- Observational health-data networks presenting federated analytical tasks.
- Applications of federated analytics relevant to public-health analytics or NCD surveillance.

Papers were considered eligible if they addressed any of the above domains in the abstracts and full text. Documents focusing only on metadata and common data models (CDM), privacy and data protection, blockchain or unrelated engineering optimization, non-health federated systems, or purely technical machine-learning benchmarking without analytical or epidemiological relevance were excluded. Editorials, proposals and study protocols were also excluded. The reason for exclusion from reading the title, abstract or full text was annotated in specific additional columns. All exclusions were double checked.

The review employed narrative and thematic synthesis.

Given the heterogeneity of study designs and the methodological objectives of the scoping review, quantitative meta-analysis was not considered.

## 3 Results

### 3.1 Overview of the literature

**Figure 1:**
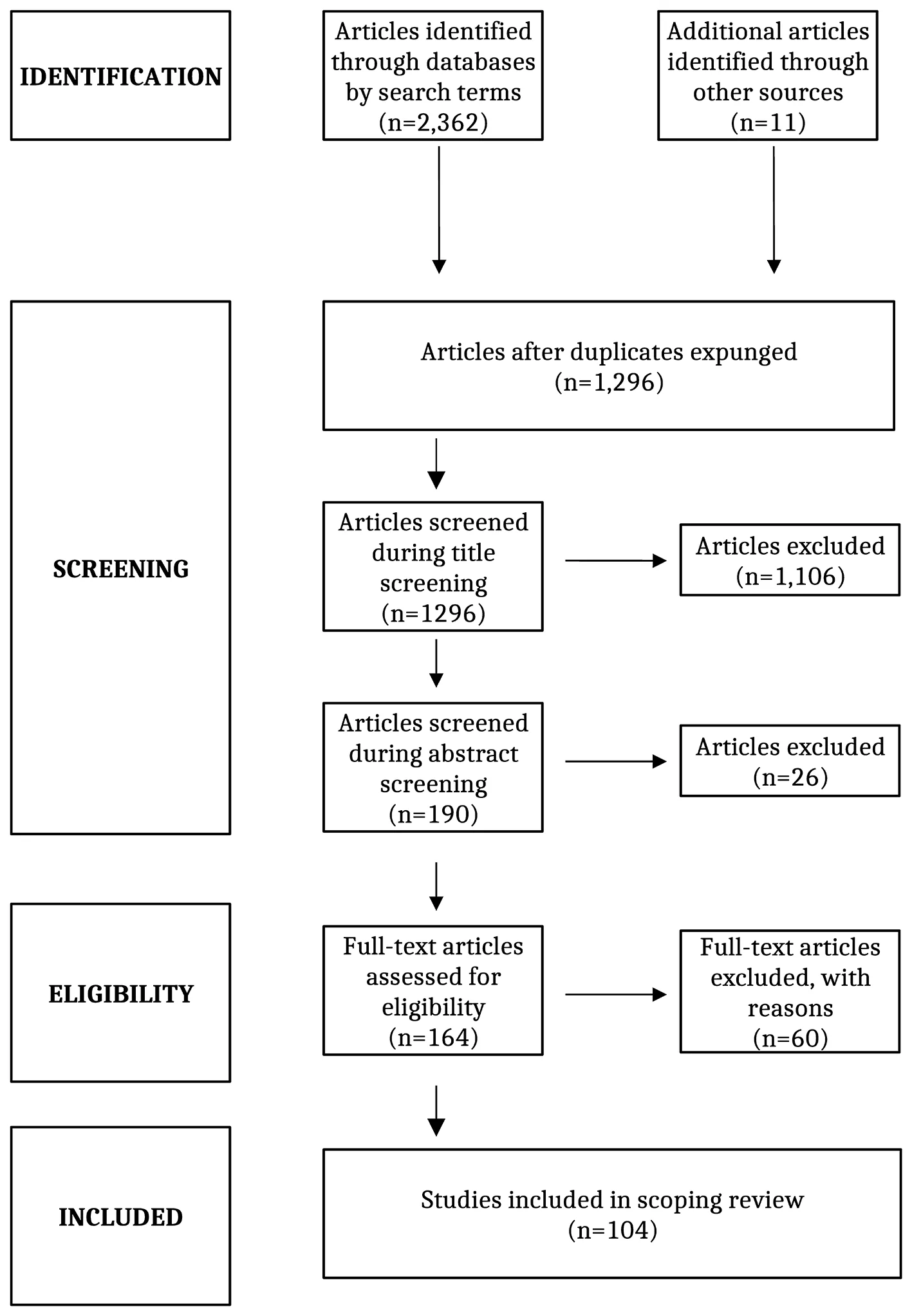
PRISMA-ScR flow diagram of the study selection process.

The results of the selection process are shown in the PRISMA-ScR flow diagram in Figure 1.

A total of 2,362 records were identified across databases. After deduplication and metadata-aware merging, 1,285 unique records remained for title and abstract screening. Additional 11 papers were added through manual retrieval. A total of 1,106 papers were excluded from reading the titles, while additional 26 were excluded from reading the abstracts and *n* = 60 from the full text.

A total of 104 references were finally extracted from the selected literature, classified according to the main categories previously identified in Table 2.

**Table 2:** Summary of Paper results (*n* = 104).

Category	Included papers
Distributed regression, including survival and longitudinal modeling	37
Federated or distributed analytical infrastructures	29
Privacy-preserving epidemiological analysis	20
Applications relevant to public-health analytics or NCD surveillance	8
Observational health-data networks presenting analytical tasks	5
Governance or interoperability infrastructures supporting secondary use of health data	5

The complete list of papers selected for the scoping review is available as Supplementary data.

Three major and partially overlapping conceptual domains emerged from the final selection:

- Distributed regression, including survival and longitudinal modeling (*n* = 37 references).
- Federated or distributed analytical infrastructures (*n* = 29 references).
- Privacy-preserving federated epidemiological analysis (*n* = 20 references).

**Figure 2:**
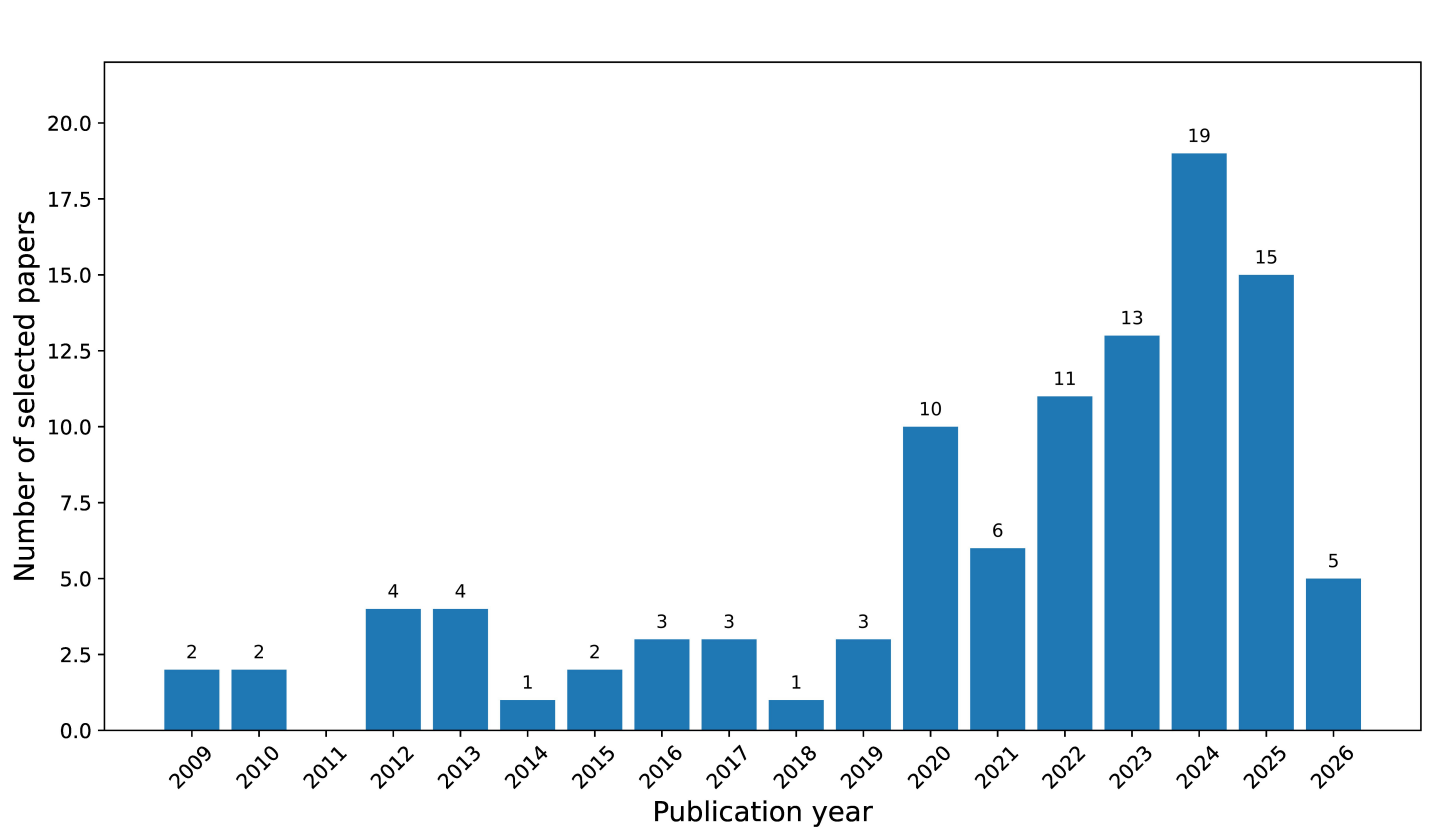
Selected papers on federated analytics by publication year (*n* = 104).

The distribution of the selected 104 publications by year is shown in Figure 2.

The literature revealed a rapidly expanding but methodologically heterogeneous ecosystem of distributed analytical approaches spanning epidemiology, biostatistics, health informatics, privacy-preserving computation, and machine learning. The terminology varied substantially across disciplines, with related approaches described as FA, distributed regression, distributed inference, privacy-preserving analytics, secure computation, federated learning, and decentralized health analytics.

Although federated learning dominated much of the broader technical literature, comparatively fewer studies addressed distributed epidemiological inference, observational analytics, or policy-grade public-health indicator production. Nevertheless, a growing body of work has emerged around distributed statistical inference frameworks to support multicentre observational research and surveillance infrastructures.

The results related to the main categories are presented in detail in the following sections.

### 3.2 Distributed regression, including survival and longitudinal modeling

The most frequent category included *n* = 37 papers including methods successfully applied in real-world studies. Across studies, distributed regression and survival-analysis frameworks consistently produced estimates closely approximating centralized pooled analyses while preserving local governance and minimizing disclosure risks.

The Grid Binary LOgistic REgression (GLORE) model integrated partial elements or non-privacy sensitive prediction values to obtain model coefficients, variance-covariance matrices, goodness-of-fit tests and ROC curves in a distributed approach (18). This approach represented one of the earliest distributed logistic-regression frameworks allowing exact pooled-equivalent estimation through iterative summary-statistic exchange without sharing patient-level data.

A major subgroup focused on communication-efficient distributed generalized linear models to reduce the overhead due to the exchange of partial results across federated nodes, starting from ODAL (One-shot Distributed Algorithm for Logistic Regression) (17). This approach enabled distributed logistic regression using only summary-statistic exchange (28). Subsequent extensions included robust heterogeneous environments (29) and Cox modeling via ODAC (One-shot Distributed Algorithm for Cox model), which covered distributed time-to-event analysis, providing good approximations of Cox proportional hazards estimation of pooled data, using only summary-level communication (30). This substantially reduced execution time from iterative distributed optimisation methods such as GLORE, making survival analysis highly suitable for large multicentric environments. Subsequent extensions further expanded ODAC, e.g. ODACT for time-varying coefficients (31) and ODACR (One-shot Distributed Algorithm for Competing Risks model), ODACoRH (One-shot Distributed Algorithm for Competing Risks model with Heterogeneity) for distributed Fine–Gray competing-risk regression across heterogeneous clinical sites (32, 33).

Distributed survival analysis and Cox modeling were also tackled by other approaches. WebDISCO (Web service for DIStributed COx model) introduced distributed Cox proportional hazards modeling without patient-level data sharing (19). VERTICOX (VERTIcally Distributed COX Proportional Hazards Model) developed vertically partitioned Cox regression (i.e. when different characteristics are stored in different data sources), using alternating direction methods of multipliers (34). WICOX (Weight-Based Integrated Cox Model) implemented integrated Cox modeling across distributed databases (35). Additional studies expanded federated modeling to federated survival forests (36), federated survival analysis with privacy-preserving release of Kaplan–Meier curves (37).

Longitudinal and causal-inference methods were included in DataSHIELD (38): GAMLSS (Generalized Additive Models for Location, Scale, and Shape) produced federated generalized additive models for location, scale and shape, enabling distributed growth and percentile modeling (39), dsSurvival 2.0 implemented privacy-enhanced distributed survival curves (40) and further routines included federated difference-in-difference analyses (41).

### 3.3 Federated or distributed analytical infrastructures

The second most frequent category included *n* = 29 papers covering the development of distributed ecosystems enabling multicentre analytics without transfer of patient-level data. Across this category, recurring themes included semantic harmonization, interoperability frameworks, distributed governance, secure analytical execution, and scalable observational infrastructures.

Several historically important European experiences emerged from projects in the area of health information, anticipating modern FA approaches and introducing the conceptual foundations of the EHDS as an evolution of epidemiological surveillance ecosystems.

Since 2005, BIRO (Best Information through Regional Outcomes) and its successor EUBIROD (European Best Information through Regional Outcomes in Diabetes) represented influential examples of governance-aware distributed epidemiological infrastructures (42). These initiatives supported harmonized diabetes surveillance across countries using common data dictionaries (43), privacy impact assessment (44–46), distributed statistical methods for the delivery of risk adjusted public health indicators and standardized reporting. The resulting infrastructure targeted population health improvement and quality of care monitoring, harmonized indicator generation and cross-national benchmarking (47). The statistical methodology was openly described in a series of public EU reports (48, 49), providing an inspiration for subsequent developments. The BIRO approach included federated logistic regression and privacy-enabled data exchange through the specification of “statistical objects,” later generalized as the “essential levels of health information” (15). These methods have been implemented in fully operational open source software integrating R, Java and PostgreSQL, with various updates (50) and types of applications (6, 20).

The DataSHIELD ecosystem represents a general solution to the problems raised by these initial experiences. This work presented a similar process to produce distributed summary statistics, using R and OPAL for data harmonization (38). Its development continued consistently over time and across different projects, extending the approach to privacy protection, secure optimization and practical FA infrastructure within a self-contained environment. DataSHIELD (Data Aggregation Through Anonymous Summary-statistics from Harmonized Individual-levEL Databases) formalized shared individual-level analysis across distributed studies (51), integrating large epidemiological cohorts within multidimensional federated infrastructures (52). The software has been framed as an ethically robust environment for multisite analysis (53), operationalising the principle of “taking the analysis to the data” across international collaborative infrastructures (16). Many studies endorsed the system, including BioSHaRE (54), integration of OMOP-CDM to support standardized federated observational analytics (55), use in the EU ORCHESTRA project infrastructure to integrate international clinical pipelines from data ingestion to FA (56) and application for modular oncology infrastructures based on FHIR interoperability standards (57). Differential privacy was also integrated into federated multitask-learning infrastructures in DataSHIELD (58).

Other relevant approaches were also developed in different areas.

In observational health-data science and pharmacoepidemiology, treatment pathways were characterized across the collaborative network OHDSI (Observational Health Data Sciences and Informatics) using distributed observational infrastructures (59). More recent approaches implemented federated pharmacoepidemiological analyses across multiple data sources (60) and conceptualized distributed data networks as core infrastructures for pharmacoepidemiology (61).

Several studies expanded federated infrastructures toward machine-learning ecosystems. CODA (Collaborative Data Analysis) was proposed as an open-source platform supporting FA and machine learning across distributed healthcare data (62).

Additional infrastructures supported real-world evidence generation and multicentre observational analytics. CO-CONNECT was developed as a federated architecture supporting distributed discovery and analytics across UK datasets (63). More recently, a European cancer-oriented data-space infrastructure integrated clinical nodes and cancer registries (64), while a federated obesity-research infrastructure was operationalised within the IMI-SOPHIA initiative (65).

Several studies addressed operational federated infrastructures designed to deploy privacy-preserving analytics in real-world environments. In particular, the Personal Health Train paradigm (66) and the infrastructure of VANTAGE6 (priVAcy preserviNg federaTed leArninG infrastructurE for Secure Insight eXchange) (67) emphasized the coordination of distributed systems within FAIR and interoperability mode.

### 3.4 Privacy-preserving federated epidemiological analysis

Additional *n* = 20 references focused specifically on reducing disclosure risks while enabling distributed epidemiological inference. Overall, this category demonstrates increasing integration of cryptographic privacy technologies, differential privacy, synthetic-data generation, and disclosure-control strategies into operational federated epidemiological ecosystems.

The DataSHIELD ecosystem dominated this category: expanded disclosure-control strategies were developed for distributed federated platforms (68), federated oncology analysis was implemented with differential privacy across hospital warehouses (69).

Cryptographic approaches were also increasingly represented, including secure multi-party computation according to GLORE (70), frameworks for federated analysis of health data using multiparty homomorphic encryption (71, 72) and privacy-preserving data quality control in federated health data networks (73).

### 3.5 Other categories

The category of “Applications relevant to public-health analytics or NCD surveillance” included *n* = 8 references focusing on operational applications of FA for public-health monitoring, chronic disease surveillance, and multicenter epidemiology. Collectively, these studies demonstrate that FA is increasingly transitioning from methodological experimentation toward operational public-health intelligence and chronic-disease analytical infrastructures.

Applications of the BIRO approach (47) were run at the international level to deliver cardiovascular indicators for the OECD project on hospital performance benchmarking (26) and a large sub-national study in diabetes, the REWINDER project of Emilia-Romagna, federating linked administrative, clinical and person-reported outcome from different local health care organizations (74).

Over the last 15 years, DataSHIELD was consistently used over time for a series of relevant applications. An international study explored the relation between road traffic noise and increased heart rate using data from three large European cohorts: LifeLines (the Netherlands), EPIC-Oxford (United Kingdom) and HUNT3 (Norway) (75). Another application pooled harmonized data from 9 birth cohorts in the EU to explore the association between pet ownership and lung function using random effects meta-analysis (76). More recently, two datasets from Canada and Austria were used to explore differences between synthetic and federated datasets (77), while a study using the Canadian Community Health Survey and European Health Interview Survey investigated the relation between risk factors and hypertension (78).

Other relevant applications proposing self-standing solutions included COVID-19 federated analytical infrastructures (79, 80).

A set of n=5 studies classified as “Observational health-data networks presenting analytical tasks” focused on operational real-world evidence generation through federated multicentric infrastructures. These studies collectively demonstrated increasing operationalisation of federated observational epidemiology across chronic disease surveillance, pharmacoepidemiology, oncology, genomics, and comparative-effectiveness research.

Distributed algorithms across research networks were used to identify clinical risk factors for opioid-use disorder (81). The DREAM TO TREAT Atopic Dermatitis Registries Collaboration implemented federated DataSHIELD analysis (82). Several studies operationalised federated observational surveillance across European infrastructures, including federated time-to-event prediction scores using heterogeneous real-world survival datasets (83).

Finally, n=5 studies classified as “Governance or interoperability infrastructures supporting secondary use of health data” emphasized governance models, metadata harmonization, interoperability frameworks, and infrastructures enabling lawful secondary use of health data. Collectively, the studies in this category framed FA as a governance-oriented infrastructural paradigm rather than solely a computational methodology.

Predictive models applied to COVID-19 adopted an OHDSI analytics pipeline for rapid development of decision support tools at patient level, showing that open source tools could be used on a routine basis for clinical decision making (84).

Additional studies addressed harmonization of heterogeneous disease registries, FHIR- and OMOP-based interoperability architectures (55), metadata governance (85) and distributed observational infrastructures (86). These studies consistently highlighted that FA depends not only on computational privacy mechanisms but also on semantic harmonization, governance alignment, interoperability standards, and institutional trust.

## 4 Discussion

The present scoping review mapped the rapidly evolving literature surrounding FA for the production of NCD indicators in the EHDS. The targeted literature identified FA as an emerging paradigm capable of reshaping the organization of public-health knowledge production within Europe.

A particularly important finding was the identification of substantial conceptual continuity between earlier European distributed epidemiological infrastructures and modern FA ecosystems.

Initiatives such as BIRO and EUBIROD were nurtured within the EU programs of health information, anticipating progress of governance-aware distributed analytics long before the emergence of federated terminology. These systems represent important historical predecessors of current EHDS-oriented FA infrastructures.

The literature suggests that distributed analytical paradigms are transitioning from experimental methodological frameworks toward increasingly operational infrastructures within real-world health-data ecosystems. The results suggest that distributed approaches may be adopted to support a broader spectrum of epidemiological functions relevant to NCD surveillance.

A taxonomy of FA approaches is shown in Table 3.

**Table 3:** Taxonomy of federated analytical approaches.

Analytical family	Representative methods	Typical analytical targets	Communication model	Key strengths	Main limitations
Distributed generalized linear models	ODAL, distributed logistic regression	Binary outcomes, risk prediction	One-shot summary statistics	Communication-efficient, near-pooled estimates	Limited handling of strong heterogeneity
Distributed survival analysis	ODAC, WebDISCO, WICOX, VERTICOX	Cox proportional hazards models	Iterative or one-shot distributed optimization	Accurate survival estimation without sharing patient data	Computational complexity for high-dimensional data
Competing-risk federated modeling	ODACoRH	Fine–Gray competing-risk regression	One-shot distributed summary exchange	Handles competing risks and heterogeneity	Limited implementations in production environments
Federated machine learning	dsMTL, FeatureCloud, federated SVMs	Prediction, imaging, classification	Iterative model-weight exchange	Scalable AI development	Often weak interpretability
Privacy-preserving infrastructures	DataSHIELD, homomorphic encryption, differential privacy	Epidemiological inference	Secure summary-statistic exchange	Strong disclosure control	Computational overhead
Observational health-data networks	OHDSI, OMOP-CDM, CO-CONNECT	Real-world evidence, comparative effectiveness	Federated cohort analysis	Large-scale multicenter epidemiology	Requires extensive harmonization
Federated longitudinal modeling	Federated GAMLSS, difference-in-differences	Growth curves, longitudinal trajectories	Distributed iterative estimation	Supports longitudinal inference	Limited methodological maturity

Across studies, DataSHIELD (16, 38, 51–53), OHDSI/OMOP infrastructures (55–57, 59), distributed observational research networks (63–65) and GLORE-based infrastructures (18) appear as relevant architectures for privacy-preserving analytics without transfer of individual-level data. The literature consistently emphasized that semantic harmonization, metadata governance, and interoperability are as important as computational technologies for the proposed purpose.

In particular, distributed statistical inference seems particularly mature for generalized regression and survival analysis. In these fields, GLORE (18) represented one of the earliest demonstrations of how pooled-equivalent distributed logistic regression could be operationalised in a federated network, using iterative exchange of summary-statistics across participating nodes. This represents an effective implementation of the early concept of “statistical object” from the BIRO approach.

ODAL (17) subsequently addressed one of the major limitations of GLORE, by dramatically reducing communication overhead through one-shot approximation strategies (29, 30). This transition from iterative distributed optimisation toward communication-efficient surrogate-likelihood methods represents a major methodological evolution in federated epidemiology.

Survival analysis was also covered by these methods. Together, GLORE and ODAL developments illustrate the progressive maturation of distributed regression methodologies from generalized linear models toward increasingly sophisticated survival and competing-risk frameworks. Other approaches for federated Cox and longitudinal models demonstrated accurate distributed time-to-event inference across multicentric environments.

At the same time, the review identified substantial asymmetry between the rapid expansion of federated-learning ecosystems and distributed epidemiological methods. Compared with the rapid expansion of federated AI and machine-learning ecosystems, comparatively fewer studies addressed generalized estimating equations, repeated-measures analysis, longitudinal inferential epidemiology, and policy-grade NCD surveillance. This gap could be closed by the evolution of future work, enabling the application of complex models to compute surveillance indicators directly on population-based data. Such evolution could close a circular loop between federated learning and analytics, using different methods interchangeably.

Another major finding concerns the convergence between FA and observational health-data networks. Studies involving large cohorts, particularly those adopting OHDSI, demonstrate that real-world evidence generation can increasingly be operationalised through distributed infrastructures (81–83). These studies collectively suggest that FA can scale up from research studies to continuous surveillance systems.

Advances in privacy preservation may further encourage the uptake from institutions handling large scale databases under their custody. Differential privacy, homomorphic encryption, disclosure-control infrastructures, synthetic-data generation and governance-aware privacy architectures are increasingly integrated into operational FA environments.

### 4.1 Federated analytics as a public-health infrastructure paradigm

The literature shows that FA should not be understood merely as a technical solution for privacy preservation, but can represent a structural reorganization of analytical infrastructures in which computation is shared between local data and centralized repositories. This transition may prove particularly relevant within the European policy context, where privacy protection, institutional autonomy, and cross-border governance remain central priorities.

An important finding of this review is the relative mismatch between the dominant federated learning discourse and the actual analytical requirements of public-health surveillance. Most public-health stakeholders are not primarily interested in training large-scale neural networks. Instead, they require transparent, reproducible, auditable, and policy-relevant statistical infrastructures capable of supporting interpretable epidemiological analyses.

Consequently, concepts such as distributed statistical inference, FA and privacy-preserving epidemiology may prove more operationally relevant than the broader federated learning paradigm, for the scope of European health policies.

Differently from federated machine learning, distributed statistical inference provides model parameters with direct epidemiological interpretation e.g. odds ratios, hazard ratios, confidence intervals and standard errors. Such outputs remain the basis of regulatory evaluation, guideline development and health-system benchmarking.

The ability to deliver parameters that are globally recognized can facilitate evaluation and independent audit, which are essential requirements for evidence-based policy making. On the other hand, although federated machine-learning models may be more accurate under specific conditions, their limited interpretability and intrinsic complexity may hamper decision-making, particularly where transparent justification of policy recommendations is required.

In this context, NCD surveillance requires analytical infrastructures capable of supporting repeated and longitudinal measurement, risk adjustment, subgroup analyses, and temporal monitoring across heterogeneous populations.

The reviewed literature suggests that federated generalized linear models and distributed regression infrastructures are already relatively mature for the production of core public health indicators in Europe. By contrast, distributed longitudinal analysis, mixed models, and survival analysis may pose methodological and operational challenges.

In particular, the complexity of distributed survival analysis appears especially relevant for cancer and cardiovascular surveillance, since Cox partial likelihood estimation depends on globally distributed risk sets that can be hard to share. Similarly, federated longitudinal models must accommodate repeated measurements, covariance estimation, and clustered observations.

The findings of this review suggest that federated infrastructures may be initially most feasible for descriptive epidemiology, distributed regression, and risk-adjusted quality indicators, before expanding toward more computationally demanding inferential frameworks.

### 4.2 Toward a standardized taxonomy of federated analytics

An important finding of this review relates to the persistent fragmentation of terminology across computer science, biomedical informatics and epidemiology. Closely related approaches are alternatively described as federated analytics, distributed inference, federated learning, privacy-preserving analytics, secure distributed computation and distributed statistical modeling, despite substantial conceptual overlap.

Future research would benefit from adopting a common taxonomy that would clearly distinguish the areas of interest of federated analytical infrastructures, distributed statistical inference, privacy-preserving analytical methods, governance and interoperability frameworks, observational federated research networks and public-health surveillance applications.

Such terminology would facilitate interdisciplinary communication while improving reproducibility and evidence synthesis.

### 4.3 Architectural, technical, operational and governance challenges

Although an in-depth evaluation of federated methods was considered beyond the scope of the review, some major architectural, technical, operational and governance challenges deserve to be outlined.

Architectural aspects span from the availability of general approaches to domain-specific applications, each presenting own strengths and weaknesses.

The review emphasized the influential role of DataSHIELD as a governance-fit model for distributed monitoring. The platform integrates disclosure control, auditing, harmonization and secure orchestration and can be safely applied in highly regulated environments. Furthermore, its evolution is continuously assured by a growing open source community.

However, the generality of the approach may also represent a major architectural weakness. In fact, the framework is tightly coupled to an R-centric client/server ecosystem in which only a pre-approved set of “ds.^*^ functions” may be successfully executed. As a result, adding new methods may be particularly complex, as they must comply with its internal disclosure rules and specific object structures. That would not change even moving to other programming languages e.g. Python.

Therefore, the framework scales less naturally to open, rapidly evolving ecosystems e.g. the EHDS vision, where interoperability and language-agnostic execution are increasingly important. In comparison, more limited tools e.g. ODAL (17), ODAC (30), ODACoR (32, 33) and WebDISCO (19) offer algorithmic primitives that can be flexibly embedded into many environments.

At the same time, the foundations of FA specified by the BIRO approach and its software prototypes for domain-specific applications, may be still useful to identify a common structure of public health information systems, applicable across chronic domains.

In this context, further technical developments should be also considered. Modern infrastructures e.g. VANTAGE6 with containerised computation, interoperability and flexible orchestration, address the separation of governance, computation and analytics more cleanly, making them easier to extend at scale (67).

New methods to reduce privacy risks deserve also attention, as gradients, score vectors, and intermediate summaries may leak sensitive information under certain conditions, particularly in small populations or highly stratified analyses. Accordingly, disclosure control procedures, minimum cell-count rules, and privacy-enhancing technologies remain essential.

Further operational challenges should be assessed, including semantic interoperability, harmonization of coding systems, denominator definition, data quality assurance, reproducibility, software versioning, asynchronous computation, and long-term sustainability (87–91).

Finally, in terms of governance-related infrastructure for the EHDS, federated infrastructures would require multilayered governance models integrating legal compliance, institutional trust, technical auditing, and reproducibility standards. To overcome these hurdles, federated architectures must be supported by solid institutional coordination and infrastructural investment.

In addition, there will need to be substantial attention toward the views and perceptions of all stakeholders, evaluating the feasibility of FA not only as a solution for international comparisons, but as a system that can efficiently federate nodes at different levels, within and across national boundaries. In this regard, this scoping review identified a range of approaches that need to be adapted to the specific actors required for their implementation, e.g. networks of disease registries and communities of people with chronic conditions.

### 4.4 Conceptual framework for federated NCD surveillance within the EHDS

**Figure 3:**
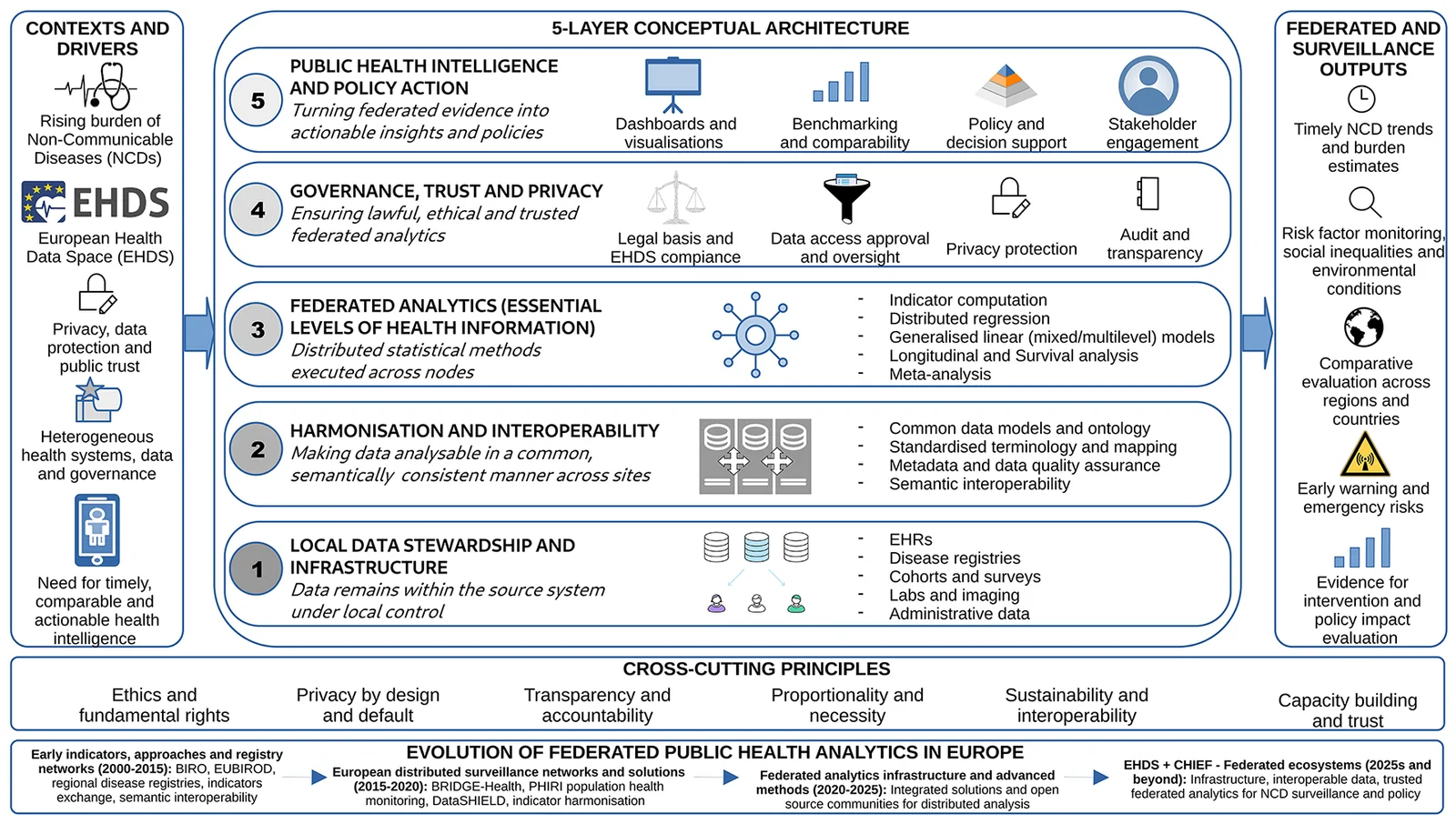
Conceptual framework for federated NCD surveillance in the EHDS.

The present review can be used to outline a conceptual framework for federated NCD surveillance within the EHDS, including five interdependent layers (see Figure 3):

1. *Local Data Stewardship*. At the foundational level, healthcare providers, regional registries, public-health institutions, and observational databases maintain within local institutional environments, the full control of different types of data sources at patient level. This layer reflects the principle of data minimization under the GDPR and preserves institutional autonomy while enabling analytical participation.
2. *Harmonization and Interoperability*. Semantic and structural interoperability mechanisms enable distributed analyses across heterogeneous data systems. Key components include common data models (e.g., OMOP), standardized vocabularies, metadata harmonization, phenotype definitions, indicator specifications, and reproducible analytical pipelines. This layer reflects lessons learned from infrastructures such as BIRO, EUBIROD, OHDSI, and DataSHIELD, all of which emphasized harmonized indicator production and interoperable analytical workflows.
3. *Federated Analytics*. The third layer includes distributed analytical engines capable of generating pooled epidemiological inference without centralized data sharing. This level specifies, through all the methods implemented e.g. distributed generalized linear models, survival analysis etc, the level of aggregation that must be maintained to deliver the requested outputs. The review suggests that future EHDS infrastructures should prioritize inferential epidemiology and surveillance-oriented analytics.
4. *Governance and Privacy*. A dedicated governance layer is required to coordinate ethical oversight, access control, legal interoperability, auditability, transparency, and privacy-preserving computation. Federated infrastructures require institutional trust frameworks and harmonized governance mechanisms.
5. *Public-Health Intelligence and Policy*. The highest level transforms distributed analytical outputs into actionable public-health intelligence. Applications addressed by this review include NCD surveillance and benchmarking of health systems performance. This layer could support the production of policy-grade indicators in the EHDS, while preserving local governance constraints.

The long-term sustainability of the framework extends beyond its governance dimension and requires sustained organizational, technical and financial commitment across all participating institutions. While federated analytics preserves local control over health data, it also entails ongoing responsibilities for maintaining secure computational nodes, software updates, semantic harmonization, metadata curation, cybersecurity, technical support and workforce development.

These operational capabilities should be regarded as core components of federated public-health infrastructures rather than temporary project activities. Within the EHDS, sustainable funding mechanisms and clearly defined institutional responsibilities will be essential to ensure that federated infrastructures evolve from research initiatives into routine services supporting continuous public-health surveillance and policy-making.

The proposed framework acknowledges the need of keeping the balance between:

- Aggregate reporting vs analytical flexibility to facilitate governance.
- Distributed regression vs local control to improve methodological integrity.
- Federated analytics vs centralized computation to enable flexible networking.
- Privacy-enhancing technologies vs direct access to strengthen data protection.

Rather than replacing centralized infrastructures, FA should likely coexist with hybrid analytical ecosystems in which different governance and computational models are selected according to specific analytical objectives and disclosure risks.

The EHDS creates an unprecedented opportunity to develop distributed public-health analytical infrastructures capable of supporting multinational NCD surveillance without requiring unrestricted centralization of individual-level data.

In this context, FA may facilitate cross-country surveillance and scalable observational studies. However, successful implementation will require coordinated development of interoperability standards, common data models, governance agreements, certification procedures, auditing infrastructures, and sustainable operational funding.

The future success of EHDS-related analytical ecosystems may depend less on sophisticated AI than on the establishment of trustworthy, reproducible, and interoperable statistical infrastructures.

The proposed conceptual framework emphasizes the need of ensuring historical continuity between a stream of initial EU public health projects and subsequent FA ecosystems that emerged in different directions. The EHDS will allow closing the loop between technical complexity and public health intelligence and policy, as recently advocated by the CHIEF expert group (20).

The findings of this review support the definition of a modular approach, which should bear upon a dynamic set of interoperable tools, consistently with the original ethos of the open source movement (92).

In this context, FA does not represent an entirely novel paradigm, but the evolution of a long standing surveillance infrastructure that can be reflected and adequately applied to solve the complexity of NCD indicators.

### 4.5 Gaps and limitations of this scoping review

This scoping review highlighted relevant gaps in the current scientific production:

- Terminology remains highly fragmented across federated learning, distributed inference, privacy-preserving analytics, biomedical informatics, and governance literatures. This fragmentation complicates evidence synthesis and may partially obscure methodological continuity between older distributed epidemiological frameworks and newer FA infrastructures.
- Much of the literature remains dominated by predictive machine-learning applications rather than inferential epidemiological methods. Distributed generalized linear modeling, survival analysis, causal inference, and longitudinal modeling remain comparatively underdeveloped.
- Relatively limited literature explicitly addresses operationalisation of federated infrastructures for policy-grade public-health analytics and NCD surveillance.

These gaps are particularly relevant to address the implementation of the EHDS, which aims to facilitate secondary use of health data across European jurisdictions while preserving privacy, governance, and institutional autonomy. In this regards, further efforts are needed to integrate interoperability, harmonization, governance standardization, methodological validation, and reproducible analytical workflows in FA.

Whereas centralized data pooling has historically dominated multi-site epidemiological analysis, federated infrastructures may increasingly become necessary in contexts characterized by governance fragmentation, cross-jurisdictional restrictions, institutional autonomy and privacy-sensitive data environments.

This review also presented several limitations that deserve to be outlined:

- Study selection and data extraction were performed by a single reviewer. Although all exclusions were subsequently rechecked to ensure internal consistency, no independent duplicate screening was conducted, which may have introduced selection bias.
- It presented an overview of the different methods for FA in relation to their applicability in the EHDS, rather than a detailed qualitative analysis of the strengths and weaknesses of different approaches.
- The terminology surrounding FA was highly heterogeneous and continuously evolving. Despite the use of a structured multi-domain search strategy, some relevant studies may have not been captured.
- The review intentionally emphasized conceptual breadth rather than exhaustive capture of novel federated-learning literature, whose evaluation was considered beyond the scope of this work.
- The review focused primarily on infrastructures and analytical paradigms relevant to the production of NCD indicators e.g. observational data and public health surveillance systems, rather than clinical predictive modeling.

## 5 Conclusions

Federated analytical infrastructures are rapidly reshaping the landscape of distributed health-data analysis. Although progressively expanding, distributed epidemiological inference remains comparatively under-represented, compared to federated learning.

The scoping review suggested that the existing infrastructures, distributed regression frameworks, and privacy-preserving computational ecosystems are mature enough to provide the essential components of cross-border federated analysis in the EHDS. Realizing this potential will require continued development of interoperable tools, methods, governance and privacy-preserving analytical workflows.

The conceptual framework outlined in this scoping review may help identifying the right synergies and planning new efforts for future research and the continuous production of timely NCD indicators in Europe.
